# An Immune Gene-Related Five-lncRNA Signature for to Predict Glioma Prognosis

**DOI:** 10.3389/fgene.2020.612037

**Published:** 2020-12-16

**Authors:** Xinzhuang Wang, Ming Gao, Junyi Ye, Qiuyi Jiang, Quan Yang, Cheng Zhang, Shengtao Wang, Jian Zhang, Ligang Wang, Jianing Wu, Hua Zhan, Xu Hou, Dayong Han, Shiguang Zhao

**Affiliations:** ^1^Department of Neurosurgery, The First Affiliated Hospital of Harbin Medical University, Harbin, China; ^2^Key Colleges and Universities Laboratory of Neurosurgery in Heilongjiang Province, Harbin, China; ^3^Institute of Neuroscience, Sino-Russian Medical Research Center, Harbin Medical University, Harbin, China; ^4^North Broward Preparatory School, Coconut Creek, FL, United States; ^5^Department of General Surgery, The First Affiliated Hospital of Harbin Medical University, Harbin, China; ^6^Department of Neurosurgery, The Pinghu Hospital of Shenzhen University, Shenzhen, China

**Keywords:** tumor immune microenvironment, immune gene sets, lncRNA, glioma, risk score

## Abstract

**Background:**

The tumor immune microenvironment is closely related to the malignant progression and treatment resistance of glioma. Long non-coding RNA (lncRNA) plays a regulatory role in this process. We investigated the pathological mechanisms within the glioma microenvironment and potential immunotherapy resistance related to lncRNAs.

**Method:**

We downloaded datasets derived from glioma patients and analyzed them by hierarchical clustering. Next, we analyzed the immune microenvironment of glioma, related gene expression, and patient survival. Coexpressed lncRNAs were analyzed to generate a model of lncRNAs and immune-related genes. We analyzed the model using survival and Cox regression. Then, univariate, multivariate, receiver operating characteristic (ROC), and principle component analysis (PCA) methods were used to verify the accuracy of the model. Finally, GSEA was used to evaluate which functions and pathways were associated with the differential genes.

**Results:**

Normal brain tissue maintains a low-medium immune state, and gliomas are clearly divided into three groups (low to high immunity). The stromal, immune, and estimate scores increased along with immunity, while tumor purity decreased. Further, human leukocyte antigen (HLA), programmed cell death-1 (PDL1), T cell immunoglobulin and mucin domain 3 (TIM-3), B7-H3, and cytotoxic T lymphocyte-associated antigen-4 (CTLA4) expression increases concomitantly with immune state, and the patient prognosis worsens. Five immune gene-related lncRNAs (AP001007.1, LBX-AS1, MIR155HG, MAPT-AS1, and LINC00515) were screened to construct risk models. We found that risk scores are related to patient prognosis and clinical characteristics, and are positively correlated with PDL1, TIM-3, and B7-H3 expression. These lncRNAs may regulate the tumor immune microenvironment through cytokine–cytokine receptor interactions, complement, and coagulation cascades, and may promote CD8 + T cell, regulatory T cell, M1 macrophage, and infiltrating neutrophils activity in the high-immunity group. *In vitro*, the abnormal expression of immune-related lncRNAs and the relationship between risk scores and immune-related indicators (PDL1, CTLA4, CD3, CD8, iNOS) were verified by q-PCR and immunohistochemistry (IHC).

**Conclusion:**

For the first time, we constructed immune gene-related lncRNA risk models. The risk score may be a new biomarker for tumor immune subtypes and provide molecular targets for glioma immunotherapy.

## Introduction

Glioma is a primary malignant tumor derived from glial cells in the central nervous system. Its annual incidence rate is 7.08 per 100,000 people, and accounts for about 75% of whole brain and other central nervous system malignancies (Lapointe et al., 2018; Ostrom et al., 2019). Clinically, gliomas are often divided into low-grade gliomas (LGGs) and glioblastomas, which have different treatment methods and prognoses. For example, LGGs are slow growing and are mainly treated by total surgical resection. The patient prognosis is relatively good (Sturm et al., 2017). However, the median survival period is less than 2 years with malignant glioblastoma progression, even with standard treatment (surgical resection, adjuvant radiotherapy, and chemotherapy) (Tan et al., 2020). In 2016, the WHO classified gliomas into five categories based on their morphology and molecular characteristics (Louis et al., 2016). Recently, immunotherapy has been used in clinical applications. However, the overall prognosis of glioblastoma patients varies greatly. This may be due to the formation of unique tumor microenvironments during long-term tumor formation and limited molecular markers that distinguish tumor subtypes (Hanahan and Weinberg, 2011). Therefore, it is important to understand the glioma immune microenvironment and screen new molecular markers, which will guide future glioma treatment.

The extracellular matrix, soluble molecules, and tumor stromal cells are the basic components of the tumor microenvironment (Cui et al., 2017). Immune cells and stromal cells are the most common non-tumor cells. Macrophages are the most abundant immune cells in brain tumors (Quail and Joyce, 2017). Glioma often recruits T cells, bone marrow-derived suppressor cells, and macrophages through several pathways to promote immune cell accumulation and transformation into different cell types (Gieryng et al., 2017). Microglia and macrophages are often activated to control anti-tumor immune responses, promote tumor cell proliferation and invasion, and achieve immune escape (Hambardzumyan et al., 2016). Human leukocyte antigen (HLA) (Machulla et al., 2001), programmed cell death-1 (PDL1) (Jackson et al., 2019), cytotoxic T lymphocyte-associated antigen-4 (CTLA4) (Nduom et al., 2015), T cell immunoglobulin and mucin domain 3 (TIM-3) (Das et al., 2017), and other immune-related genes participate in the immune escape process. Therefore, treatments targeting immune checkpoints, microglia, and macrophages are used in the clinic (Poon et al., 2017). However, some patients are in a state of immune tolerance. To improve the quality of medical care and increase the understanding of the immune microenvironment, tumor immune gene analysis is common. Considering tumor-associated immune genes, investigating immune gene sets with guided evolutionary simulated annealing (GESA) can more comprehensively reflect the glioma immune microenvironment *in vivo* to better establish a prognostic model, find effective molecular markers, and perform effective targeted treatment (Molinaro et al., 2019).

With the development of high-throughput technology and the establishment of public databases, the molecular understanding of tumors has rapidly developed (Serratì et al., 2016), leading to improved understanding of tumor pathogenesis and improved biomarker screening. Importantly, some long non-coding RNA (lncRNA) has been identified as potential glioma biomarkers (Peng et al., 2018). Previously, lncRNAs were hypothesized to have no coding function and were regarded as transcriptional noise. However, lncRNAs play an important regulatory role in gene transcription and post-transcriptional modification. Indeed, lncRNA can regulate inflammation and participate in immune gene expression, thus affecting the tumor immune microenvironment (Chen, 2016; Mathy and Chen, 2017). For example, lincRNA-Cox2 regulates chromatin complex remodeling and participates in inflammatory gene expression (Hu et al., 2016). lncRNA nuclear-enriched abundant transcript 1 (NEAT1) participates in the regulation of interleukin (IL)-8 transcription, thus affecting cytokine response, and induces immune gene expression (Hirose et al., 2014). High HOTAIR lncRNA expression promotes the secretion of monocyte chemoattractant protein-1 (MCP-1/CCL2) by tumor cells and promotes the proliferation of tumor-associated macrophages (TAM) and myeloid-derived suppressor cells (MDSC) in the immune microenvironment (Botti et al., 2019). The complex relationship between lncRNAs and the tumor immune microenvironment has been gradually revealed, and the mechanism of immune-related lncRNA in a variety of tumors has been reported (Hu et al., 2019). However, the relationship between lncRNAs and the glioma immune environment remains unclear.

We analyzed glioma samples downloaded from The Cancer Genome Atlas (TCGA) and Chinese Glioma Genome Atlas (CGGA), to examine the glioma immune microenvironment using the single-sample GSEA method. Then, we screened lncRNAs related to the analyzed immune gene set. Using survival curve and Cox regression analysis, a five-lncRNA prognosis model related to the immune gene set was constructed, and the relationship between the risk score and the glioma patient prognosis was explored. Our results provide new ideas for the clinical immunotherapy of glioma.

## Materials and Methods

### Patient and Glioma Samples

This study was approved by the patients and the Ethics Committee of the First Affiliated Hospital of Harbin Medical University. All glioma tissue samples were obtained from the surgical resection tissue of glioma patients (*n* = 18); non-tumor brain tissue was used as the negative control group (*n* = 5). Tissue samples are stored separately in liquid nitrogen and paraffin embedded.

### Data Extraction

Sequencing data collected from glioma patients were downloaded from public databases. We excluded samples with incomplete clinical information. In total, we downloaded 697 (168 GBM, 529 LGG) glioma RNA-seq and 669 (510 LGG, 159 GBM) clinical sample information datasets from the TCGA database^1^, 1018 (375 GBM, 643 LGG) glioma RNA-seq and 971 (596 LGG, 375 GBM) clinical sample information datasets from the CGGA database^2^ (Jiang et al., 2016; Hu et al., 2018), and 1152 normal brain RNA-seq datasets from the Genotype-Tissue Expression (GTEX) database^3^ (GTEx Consortium, 2015).

### Immune Grouping and Correlation Analysis

In the single-sample GSEA method, each sample was scored according to 29 immune gene sets and divided into three groups by hierarchical clustering (Molinaro et al., 2019). We used Estimate package to calculate the tumor microenvironment indicators for each sample and analyze the tumor purity (Yoshihara et al., 2013). Then, we used the R-x64-4.0.2 language package to analyze the three immune-related gene and patient prognosis groups. Finally, we analyzed immune cell infiltration in each tumor sample using the CIBERSORT method (Newman et al., 2015) (*p* < 0.05).

### Risk Model

Nine lncRNAs were screened based on the correlation between identified lncRNAs and the immune gene sets (*R*^2^ ≥ 0.62) in CGGA. An additional five prognosis-related lncRNAs were identified using univariate and multivariate survival analyses by Cox regression model (Malinchoc et al., 2000). We divided the samples from the CGGA database into high- and low-risk groups according to the median risk score (Risk score = correlation_lncRNA1 × expression_lncRNA1 + correlation_lncRNA2 × expression_lncRNA2 + correlation_lncRNAn × expression_lncRNAn) (Chen et al., 2007; Zhang et al., 2020b). Survival curve and Cox regression analysis were used to construct the immune gene set-related lncRNA risk model.

### Risk Model Assessment

We used cor.test function to detect the relationships between lncRNAs (Zhang et al., 2020a). Then, we evaluated the accuracy of the risk model using univariate, multivariate, and receiver operating characteristic (ROC) curves. ggpubr package was used to show the relationship between lncRNAs, clinical symptoms, and immune status. Then, we use principal component analysis for model clustering through scatterplot3d package (Ma and Dai, 2011).

### GSEA for Enrichment Analysis

We used clusterProfiler, colorspace, and enrichplot package to perform GO and KEGG analysis based on the sequence of genes which was sorted each gene in descending order of log2FoldChange [log2 (Mean of high immune group genes/Mean of low immune group genes)], and drew a bubble chart (*p* < 0.05) through ggplot2 package (Cheng et al., 2019).

### Quantitative RT-PCR (qRT-PCR)

Total RNA was prepared using TRIzol Reagent (Invitrogen, Carlsbad, CA, United States) according to the manufacturer’s instructions. The concentration of the total RNA was detected by NanoDrop 2000 (Thermo Scientific^TM^). Total RNA (1000 ng) was reverse transcribed into cDNA using qPCR RT Kit (TOYOBO, Japan). Relative expression of target gene to the housekeeping gene GAPDH was determined by qRT-PCR using FastStart Universal 96 SYBR Green Master (ROX) (Roche, Germany). All primer sequence used in this study is listed in Supplementary Table 1. Analysis between the two groups was performed by an unpaired *t*-test; *P* < 0.05 was considered statistically significant.

### Immunohistochemistry (IHC)

The tissue sample immersed in formalin is wrapped in paraffin and sliced into 5 μm thick sections. Then sample sections were incubated for PDL1, CTLA4, CD3, CD8, and INOS primary antibodies at 4°C overnight and secondary antibodies at 37°C for 30 min. Next, samples were visualized by using the diaminobenzidine (DAB) substrate kit for 10 min. After intensive washing, samples were counterstained with hematoxylin, then dehydrated and coverslipped according to manufacturer’s protocol. The results of immunohistochemistry (IHC) were taken with Leica microscope.

### Statistical Analysis

All analyses were performed with GraphPad Prism 7, R version 3.6.1 and corresponding packages. For all data, the statistical significance is: ^∗^*P* < 0.05, ^∗∗^*P* < 0.01, ^∗∗∗^*P* < 0.001.

## Results

### The Tumor Immune Microenvironment Reflects Tumor Purity

**FIGURE 1:**
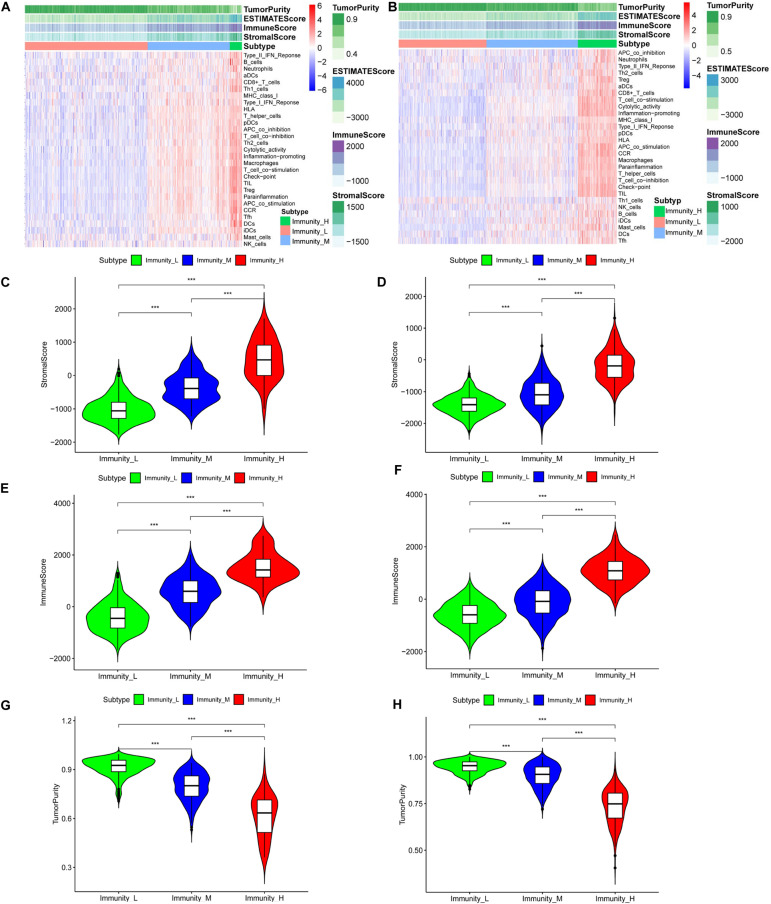
The tumor immune microenvironment is related to the expressed immune genes. Heatmaps of the tumor immune microenvironment in the TCGA **(A)** and CGGA **(B)** datasets. Violin plots of the stromal cell scores among immune groups in the TCGA **(C)** and CGGA **(D)** datasets. Violin plots of the immune scores among the immune groups in the TCGA **(E)** and CGGA **(F)** datasets. Violin plots of tumor purity in the TCGA **(G)** and CGGA **(H)** datasets, ***P < 0.001.

Normal brain tissue maintains a low-medium immune state, while gliomas are clearly divided into low-immunity groups (immunity_L), medium-immunity groups (immunity_M), and high-immunity groups (immunity_H) (Supplementary Figure 1A and Figures 1A,B). From immunity_L to immunity_H, the stromal score, immune score, and estimate score (stromal score combined with immune score) increase, and the tumor purity decreases. We further quantified different immunity groups scores and drew violin plots. The changes of immune stromal cells in the tumor microenvironment and the decrease in tumor purity are consistent with Figures 1C,E, Supplementary Figure 1B, Figure 1G (TCGA, *p* < 0.001), Figures 1D,F, Supplementary Figure 1C, and Figure 1H (CGGA, *p* < 0.001). In order to better understand the tumor microenvironment and find potential therapeutic targets, whether there are differences in immune-related genes is worthy of our further study.

### Immune Gene Expression in the Three Groups

**FIGURE 2:**
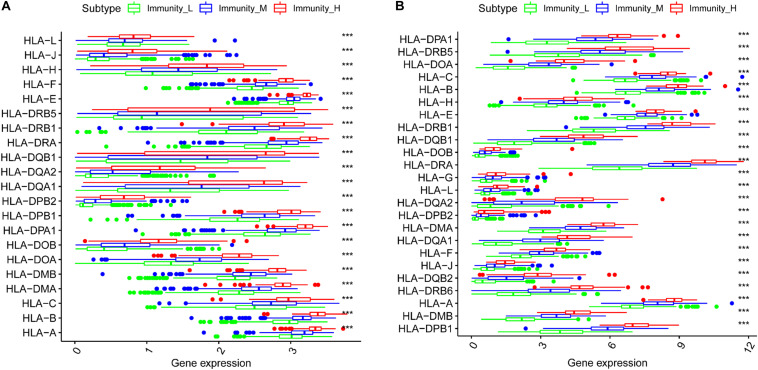
The correlation between HLA-related genes and immune groups. **(A)** TCGA. **(B)** CGGA.

**FIGURE 3:**
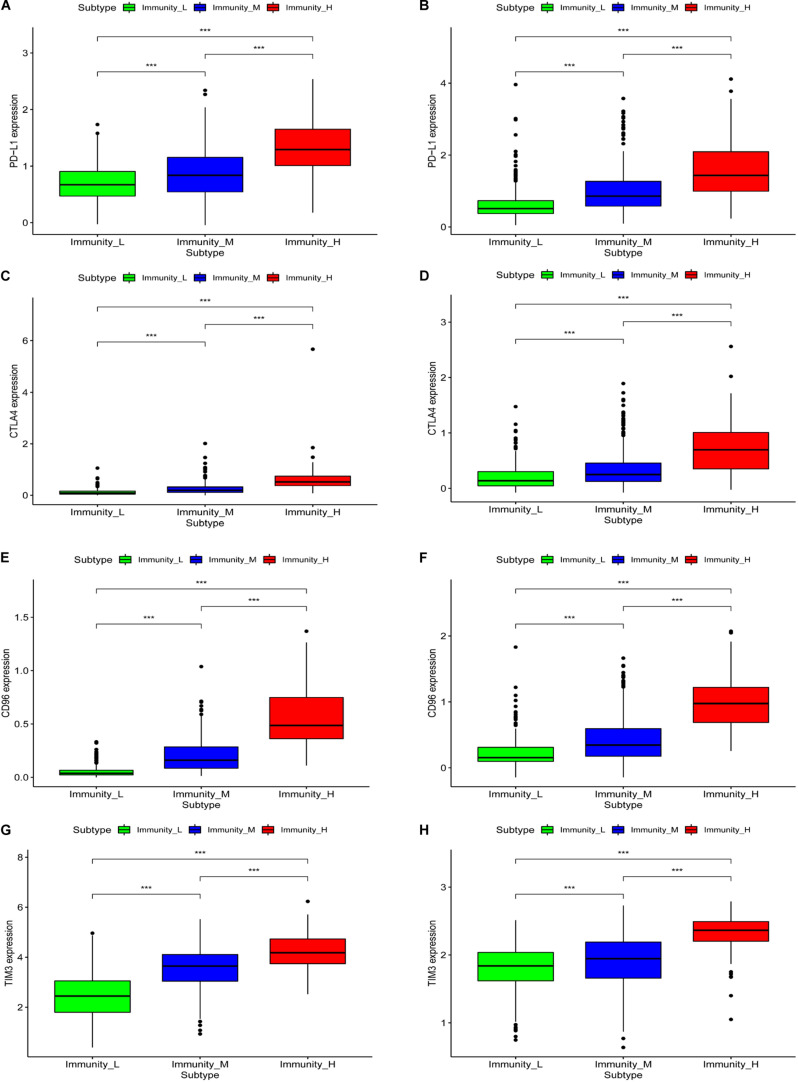
The correlation between immune groups and immune checkpoints. PDL1 expression is based on TCGA **(A)** and CGGA **(B)**, CTLA-4 expression is based on TCGA **(C)** and CGGA **(D)**, CD96 expression is based on TCGA **(E)** and CGGA **(F)**, TIM-3 expression is based on TCGA **(G)** and CGGA **(H)**, ***p < 0.001.

We generated boxplots to evaluate the expression of immune-related genes during the immune response. As shown in Figures 2A,B, HLA-related gene expression gradually increased from the immunity_L to immunity_H groups (*p* < 0.001). We also found that PDL1 (Figure 3A, TCGA; Figure 3B, CGGA), CTLA4 (Figure 3C, TCGA; Figure 3D, CGGA), CD96 (Figure 3E, TCGA; Figure 3F, CGGA), TIM-3 (Figure 3G, TCGA; Figure 3H, CGGA), and CD276 (Supplementary Figure 1D, TCGA; Supplementary Figure 1E, CGGA) expression levels also increased from the immunity_L to immunity_H groups. However, HLA-related gene expression promotes immune responses to clear tumors, while immune checkpoint genes (PDL1, CTLA4, TIM-3, and CD276) suppress immune responses and facilitate tumor proliferation and metastasis. Therefore, we further analyzed patient outcomes.

### Patient Prognosis in the Different Immune Groups

**FIGURE 4:**
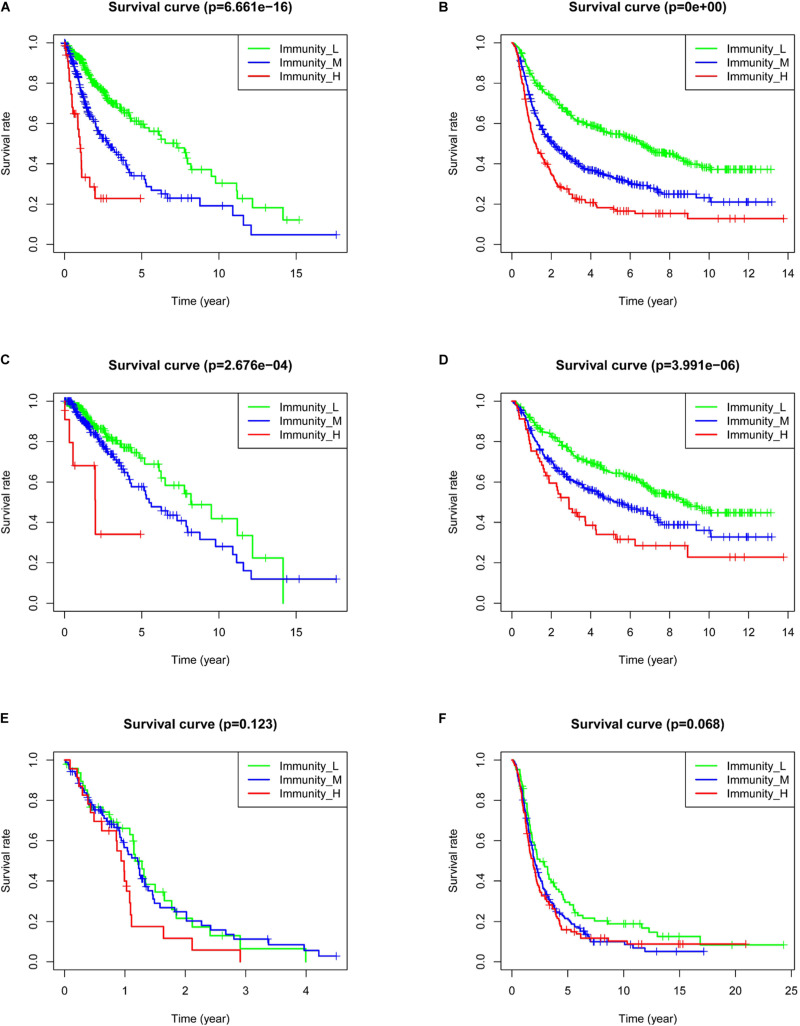
Correlation between immune grouping and survival time of glioma patients. **(A)** TCGA and **(B)** CGGA in all glioma patients. **(C)** TCGA and **(D)** CGGA in low-grade glioma patients. **(E)** TCGA and **(F)** CGGA in GBM patients.

To analyze the effect of gene expression on patient prognosis in the different immune groups, we drew survival curves for the TCGA (669 samples: 510 LGG and 159 GBM samples) and the CGGA (971 samples: 596 LGG and 375 GBM samples) (Figure 4). Among the glioma patients, patients in the immune_L group had the best prognosis, followed by the immune_M group, and the immune_H group had the worst prognosis (Figure 4B, *p* < 0.001). Among LGGs, prognosis in the different immune groups was similar (Figures 4C,D, *p* < 0.001). In GBM, the prognosis in the immune_H group tended to be worse than in the immune_L group, but the difference was not statistically significant (*p* > 0.05).

### Risk Models of Five lncRNAs Related to the Immune Gene Sets

**FIGURE 5:**
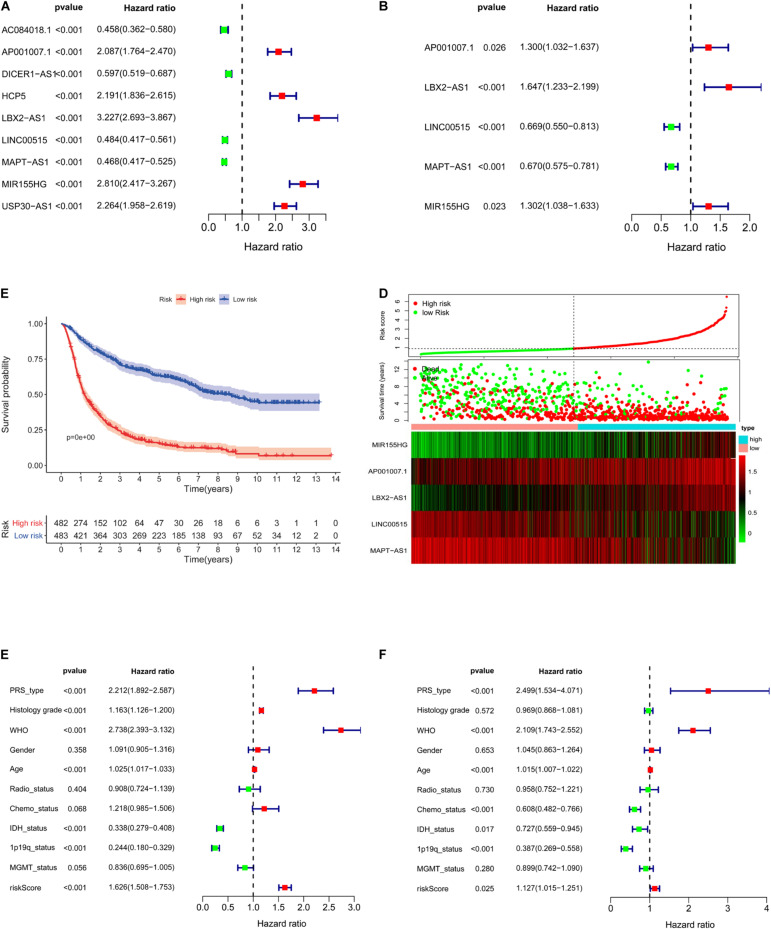
Construction of a five-lncRNA risk model based on CGGA. Univariate (**A**, *p* < 0.001) and multivariate (**B**, *p* < 0.001) survival model analysis of lncRNA related to immune gene set. Survival curves of glioma patients with different risk factors (**C**, *p* < 0.001). The risk curve of five-lncRNA model **(D)**. Univariate (**E**, *p* < 0.001) and multivariate analysis (**F**, *p* < 0.025) of multiple clinical indicators of the risk model.

**FIGURE 6:**
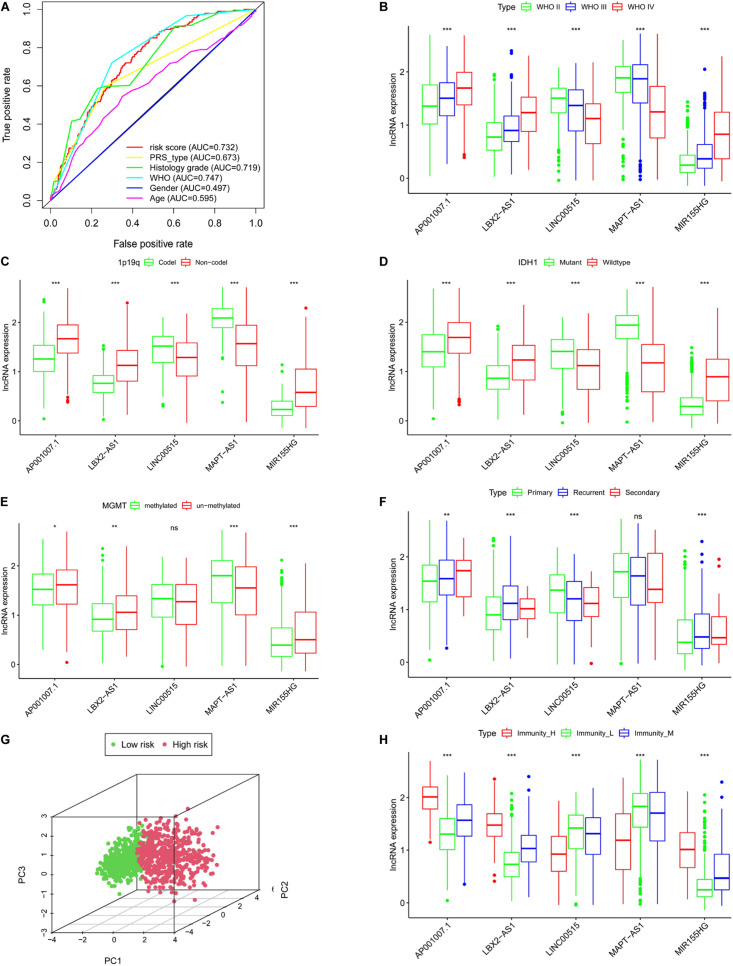
The clinical characteristics of the risk model are based on CGGA. **(A)** Roc curves of multiple clinical indicators. WHO grade **(B)**, 1p19q status **(C)**, IDH status **(D)**, MGMT methylation status **(E)**, PRS type **(F)**. **(G)** Principal component analysis of lncRNA related to immune gene set. **(H)** The expression of lncRNAs in different immune groups, *P < 0.05, **P < 0.01, ***P < 0.001, ns: not statistically significant.

Nine lncRNAs were screened based on their coexpression with immune-related genes. The nine lncRNAs we screened were AC084018.1, AP001007.1, DICER1-AS1, HCP5, LBX2-AS1, LINC00515, MAPT-AS1, USP30-AS1, and MIR155HG. After univariate (Figure 5A) and multivariate (Figure 5B) analyses, AP001007.1, MIR155HG, and LBX2-AS1 were identified as independent risk factors [hazard ratio (HR) > 1, *P* < 0.05], and LINC00515 and MAPT-AS1 were identified as independent protective factors (HR < 1, *P* < 0.001). All the lncRNAs were related to prognosis in CGGA-mRNAseq_325 and CGGA-mRNAseq_625 samples (Supplementary Figure 2, *p* < 0.001). Then, five lncRNAs (AP001007.1, MIR155HG, LBX2-AS1, LINC00515, and MAPT-AS1) were used to construct a risk model and draw survival (Figure 5C, *p* < 0.001) and risk curves (Figure 5D). The results show that as the patient risk increases, the survival time decreases, and the overall death rate increases. Finally, correlation analysis showed that the primary, recurrent, and secondary (PRS) type, World Health Organization (WHO) grade, isocitrate dehydrogenase (IDH)-mutant, 1p/19q co-deleted, age, and risk score were independent prognostic factors (Figures 5E,F, *p* < 0.05). Importantly, the risk score [area under the curve (AUC) = 0.732] and WHO (AUC = 0.747) had potential diagnostic value (Figure 6A). Thus, our risk model has clear diagnostic value.

### Clinical Characteristics of the Five lncRNAs

We next clarified the correlation between lncRNAs and clinical characteristics based on CGGA database. The results indicated that as the WHO level increased, AP001007.1, LBX-AS1, and MIR155HG expression also increased, while MAPT-AS1 and LINC00515 expression decreased (Figure 6B, *p* < 0.001). In addition, 1p19q no-codeletion (Figure 6C), IDH1 wildtype (Figure 6D), MGMT un-methylated (Figure 6E), and recurrent glioma (Figure 6F) compared with 1p19q deletion (Figure 6C), IDH1 mutant (Figure 6D), MGMT methylated (Figure 6E), and primary glioma (Figure 6F), AP001007.1, LBX-AS1, and MIR155HG also was high expression, while MAPT-AS1 and LINC00515 were also low in CGGA (except for LINC00515 in Figure 6E and MAPT-AS1 in Figure 6F, *p* > 0.05). Then, principal component analysis also showed that the risk model could divide the high- and low-risk groups into different subgroups (Figure 6G).

### The Correlation Between lncRNA and Immunity

**FIGURE 7:**
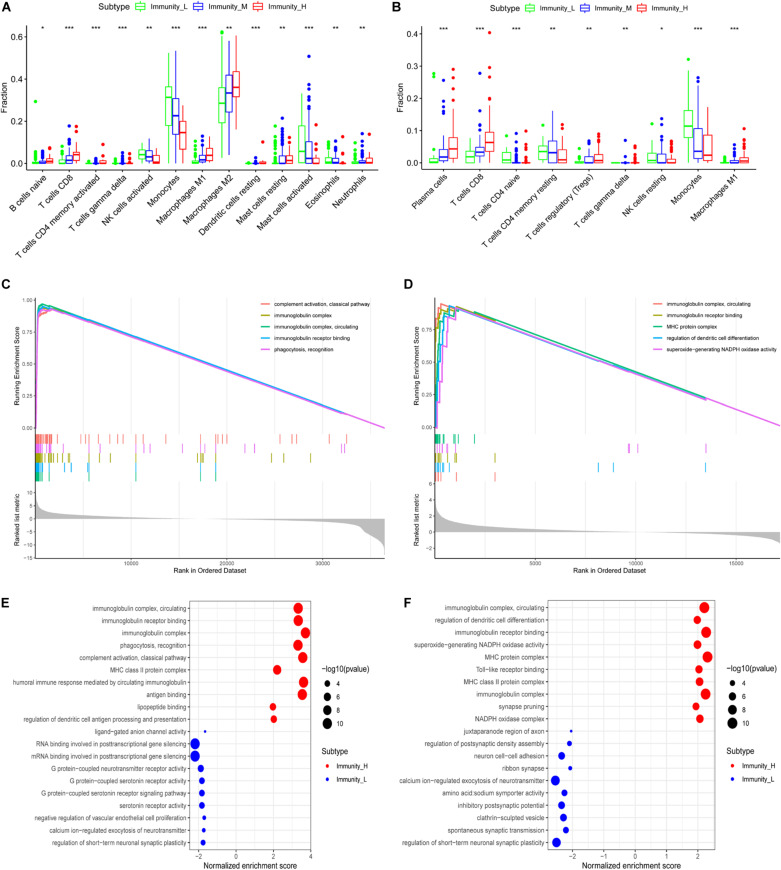
Functional enrichment analysis of genes related to immune gene set by GSEA. The correlation between immune grouping and infiltrating immune cells is based on TCGA **(A)** and CGGA **(B)**. The GO analysis of differential genes is in TCGA **(C)** and CGGA **(D)**, and the results are visualized in TCGA **(E)** and CGGA **(F)**, *P < 0.05, **P < 0.01, ***P < 0.001.

Using correlation analysis, we found that the lncRNAs in the risk model are associated (Supplementary Figure 3A). The risk score is closely related to the lncRNAs, PDL1, TIM-3, and B7-H3 (Supplementary Figures 3B–I). In addition, we found that AP001007.1, LBX2-AS1, and MIR155HG had the highest expression, while MAPT-AS1 and LINC00515 expression was the lowest in the immune_H group. In contrast, the expression of AP001007.1, LBX-AS1, and MIR155HG were relatively low, while the expressions of MAPT-AS1 and LINC00515 were relatively high in the immune_L group (Figure 6H). We next analyzed the immune-infiltrating cells in each group. In the immune-H group, we found that naive B cells, plasma cells, CD8 + T cells, regulatory T cells (Tregs), M1 macrophages, M2 macrophages, resting mast cells resting, and infiltrating neutrophils increased. CD4 + naive T cell, inactivated CD4 + memory T cell, monocyte, inactivated natural-killer (NK) cell, and activated NK cell infiltration decreased (Figure 7A, TCGA; Figure 7B, CGGA *p* < 0.05).

### GO Enrichment and KEGG Pathway Analysis

**FIGURE 8:**
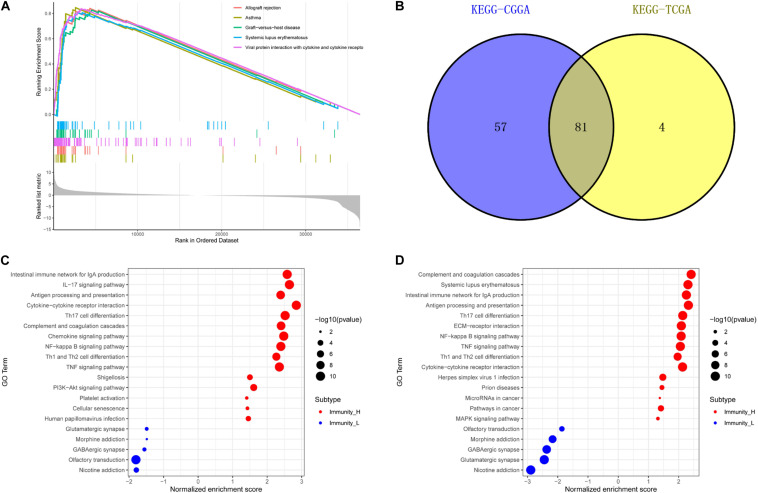
Function enrichment of genes related to immune gene set by GSEA. **(A)** KEGG in TCGA. **(B)** The intersection of related pathways is based on TCGA and CGGA. The bubble chart of the enrichment pathway is in TCGA **(C)** and CGGA **(D)**.

We used GSEA to analyze enriched differential genes in the immune_H and immune_L groups. We observed that the differential genes were enriched in immunoglobulin complex, circulating, immunoglobulin receptor binding, and MHC protein complex (Figures 7C–F). Further KEGG function analysis (Figure 8A and Supplementary Figure 3J) showed allograft rejection, asthma, intestinal immune network for IgA production, and cytokine–cytokine receptor interaction may be activated cell signaling pathways. The intersection of the two data sets revealed 81 cell signal pathways involved in glioma (Figure 8B). The five lncRNA we identified may regulate the immune microenvironment through cytokine–cytokine receptor interaction, antigen processing and presentation, complement and coagulation cascades, and intestinal immune network for IgA production (Figures 8C,D).

### *In vitro* Validation of the Risk Model

**FIGURE 9:**
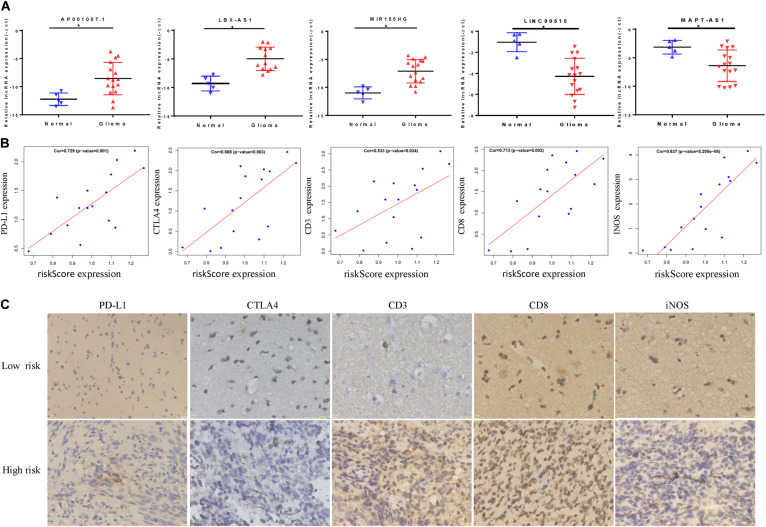
*In vitro* experiment based on riskScore model. **(A)** The abnormal expression of five lncRNAs in gliomas was confirmed by qRT-PCR. **(B)** The correlation between riskScore and immune indicators is verified by qRT-PCR. **(C)** Immunohistochemical results of patients in different risk groups, *P < 0.05.

Through qRT-PCR, we confirmed that AP001007.1, MIR155HG, and LBX2-AS1 are highly expressed, while LINC00515 and MAPT-AS1 are low expressed in gliomas compared to the control group (Figure 9A). Then, we further found that the risk score was positively correlated with the expression of PDL1, CTLA4, CD3, CD8, and INOS (Figure 9B, cor > 0.5). Finally, the immunohistochemical results also confirmed that the expression of PDL1, CTLA4, CD3, CD8, and INOS in the high risk group was significantly higher than low risk group base on protein level (Figure 9C).

## Discussion

Glioma cells form a complex regulatory network via the extracellular matrix, stromal cells, and infiltrating immune cells (Hanahan and Coussens, 2012). Some cells secrete factors and lncRNAs to promote inflammation and angiogenesis in tumors, thereby promoting malignant tumor progression and immune escape (Pitt et al., 2016; Dagogo-Jack and Shaw, 2018). It is critical to understand the tumor immune microenvironment and screen new markers to enable targeted glioma therapy for glioma. Abundant research on immune cells has been performed (Charoentong et al., 2017; Jia et al., 2018). However, the cell types, functions, and pathways associated with glioma remain unclear. Therefore, we analyzed 1715 glioma and 1152 normal brain tissue samples using the single-sample GSEA method. We found that the immune environment in gliomas was very different from the immune environment in normal brain tissues. In the immune_H group, the tumor immune cell, and stromal cell content increases, the tumor purity decreases, and tumor heterogeneity becomes greater. These conclusions are in line with previous findings (Hanahan and Coussens, 2012; Pitt et al., 2016; Dagogo-Jack and Shaw, 2018) indicating that this method can accurately reflect the basic conditions of the tumor microenvironment.

Human leukocyte antigens and immune checkpoints are an indispensable regulator of the immune microenvironment (Topalian et al., 2016; Pereira et al., 2019). In the immune_H group, PDL1, CTLA4, TIM-3, and CD96 expression were increased. Immune checkpoints act to negatively regulate immune regulation. Normal immune surveillance and cell killing ability are weakened in many tumors. Further, tumors often have immune escape or immunotherapy resistance mechanisms, leading to ineffective clinical treatment (Field et al., 2017; Qian et al., 2018). We also observed that among all the glioma samples, the immune_H group had the worst prognosis, followed by the immune_M group, and the immune_L group had the best prognosis. This conclusion also supports previous results. Moreover, GSEA has been used in many studies and has a certain degree of credibility (Ma et al., 2020; Wang et al., 2020). Therefore, the single-sample GSEA method based on expressed immune genes can distinguish the biological characteristics of the immune microenvironment between different gliomas, which provides the possibility for screening the immune microenvironment-related biomarkers.

Long non-coding RNAs can regulate the tumor immune microenvironment and can be used as biomarkers. For example, NF-kappaB interacting lncRNA (NKILA) can promote the immune escape of tumor cells by regulating T cell activity (Huang et al., 2018). Further, SATB2-AS (the antisense transcript of SATB2 protein) can directly combine with WDR5 (WD repeat containing protein 5) and GADD45A (growth arrest and DNA damage protein 45A) to regulate SATB2 expression, thereby inhibiting tumor cell metastasis and regulating the tumor immune microenvironment (Xu et al., 2019). Immune-related lncRNAs have carcinogenic effects in several tumors, and can be used as biomarkers (Li et al., 2020). Thus, it is very important to determine whether lncRNAs related to the immune gene set have clinical diagnostic and prognostic value. We screened five lncRNAs using the Cox regression method and constructed a prognostic model. We found that the risk score is related to prognosis and is an independent factor that can be used for clinical diagnosis. We further observed that five lncRNAs interact and are closely related to the clinical symptoms of glioma patients (WHO grade, IDH1 status, 1q19q status, and MGMT). Principle component analysis (PCA) analysis showed that subgroups within the high- and low-risk groups can be well distinguished using our method. These conclusions show that the risk scores of the five lncRNAs related to the immune gene set can predict patient prognosis and clinical characteristics, and can be used as a new biomarker to inform clinical diagnosis and treatment.

We used boxplots to visualize lncRNA expression in each immune group. In the immune_H group, we found that AP001007.1, LBX2-AS1, and MIR155HG were highly expressed, while LINC00515 and MAPT-AS1 expression was low. The immune_L group showed the opposite trend. Survival analysis showed that AP001007.1, LBX2-AS1, and MIR155HG were risk factors, and their high expression predicted poor patient outcomes. LINC00515 and MAPT-AS expression were protective indicators, and low expression predicted poor patient prognosis. LBX2-AS1 produces malignant behavior in gliomas by conferring resistance to cell apoptosis (Chen et al., 2020). MIR155HG promotes immune cell infiltration and immune resistance (Peng et al., 2019). In contrast, MAPT-AS1 expression indicates a good prognosis for cancer patients (Wang et al., 2019). Therefore, the immune gene set-related model we constructed has considerable credibility.

We found that the five lncRNAs we analyzed may promote immune cell infiltration through cytokine–cytokine receptor interaction, antigen processing and presentation, complement and coagulation cascades, and may contribute to immune resistance and tolerance, ultimately leading to poor patient prognosis. However, this study also has some limitations. First, because there was no information regarding MGMT, 1p19q, and other related molecules in the TCGA dataset, only a single CGGA cohort was used for statistical analysis. The CGGA sample information is mainly clinical sample information from Chinese patients, which may only indicate region-specific effects. Second, basic experiments have also verified the important role of some immune gene-related lncRNAs in regulating glioma development. However, the mechanism underlying our prognostic model remains unclear and requires additional experiments to verify our *in silico* results. Third, we confirmed that the five lncRNA have potential clinical value to identify risk factors, but more factors should be considered, especially considering multimodal glioma development.

## Conclusion

Immune-related genes can reflect the characteristics of the immune microenvironment. To reveal the mechanism of partial resistance or treatment resistance within a new risk model, five immune-related lncRNAs were analyzed and shown to have good stability and feasibility (AP001007.1, LBX-AS1, MIR155HG, MAPT-AS1, and LINC00515). Thus, our study reveals biomarkers that distinguish specific glioma groups and can be used in the clinical diagnosis and treatment of glioma.

## Data Availability Statement

The public databases mined in the study are included in the article/Supplementary Material. Further inquiries can be directed to the corresponding author/s.

## Ethics Statement

This study was approved by the Ethics Committee of the First Affiliated Hospital of Harbin Medical University.

## Author Contributions

XZW, MG, JYY, and QY collated and analyzed the data. CZ, QYJ, STW, DYH, XH, and LGW completed the writing and repair of the manuscript. JZ, HZ, JNW, and SGZ designed and guided the subject. All authors reviewed and approved the final manuscript.

## Conflict of Interest

The authors declare that the research was conducted in the absence of any commercial or financial relationships that could be construed as a potential conflict of interest.

## Footnotes

1 https://www.cancer.gov/tcga

2 http://www.cgga.org.cn/

3 https://commonfund.nih.gov/GTex

## Supplementary Material

Supplementary Figure 1 **(A)** The heatmap of the immune microenvironment of normal brain tissue base on GTEX. The relationship between immune grouping and ESTIMATEScore in TCGA **(B)** and CGGA **(C)**. The correlation between CD276 and immune grouping in TCGA **(D)** and CGGA **(E)**.

Supplementary Figure 2 Survival curve of glioma patients in CGGA mRNAseq_693 and CGGA mRNAseq_325. **(A)** ap0001007.1, **(C)** LBX2-AS1, **(E)** LINC00515, **(G)** MAPT-AS1, **(I)** MIR155HG in CGGA mRNAseq_325. **(B)** ap0001007.1, **(D)** LBX2-AS1, **(F)** LINC00515, **(H)** MAPT-AS1, **(G)** MIR155HG in CGGA mRNAseq_325 database.

Supplementary Figure 3 Correlation analysis of risk score, lncRNA, and immune checkpoint. **(A)** Correlation analysis of lncRNAs related to immune gene set. **(B)** KEGG in CGGA. **(C)** Analysis of the correlation between risk scores and lncRNAs or immune checkpoints in **(B)** LINC00515, **(C)** MIR155HG, **(D)** LBX2-AS1, **(E)** MAPT-AS1, **(F)** ap0001007.1 or **(G)** TIM-3, **(H)** B7-H3, **(I)** PDL1, **(J)** KEGG in CGGA.

## References

[B1] Botti G. Scognamiglio G. Aquino G. Liguori G. Cantile M. (2019). LncRNA HOTAIR in tumor microenvironment: what role? *Int. J. Mol. Sci.* 20:2279. 10.3390/ijms20092279 31072041 PMC6539022

[B2] Charoentong P. Finotello F. Angelova M. Mayer C. Efremova M. Rieder D. (2017). Pan-cancer immunogenomic analyses reveal genotype-immunophenotype relationships and predictors of response to checkpoint blockade. *Cell Rep.* 18 248–262. 10.1016/j.celrep.2016.12.019 28052254

[B3] Chen H. Y. Yu S. L. Chen C. H. Chang G. C. Chen C. Y. Yuan A. (2007). A five-gene signature and clinical outcome in non-small-cell lung cancer. *N. Engl. J. Med.* 356 11–20. 10.1056/NEJMoa060096 17202451

[B4] Chen L. L. (2016). Linking long noncoding RNA localization and function. *Trends Biochem. Sci.* 41 761–772. 10.1016/j.tibs.2016.07.003 27499234

[B5] Chen Q. Gao J. Zhao Y. Hou R. (2020). Long non-coding RNA LBX2-AS1 enhances glioma proliferation through downregulating microRNA-491-5p. *Cancer Cell Int.* 20:411. 10.1186/s12935-020-01433-2 PMC7448496 32863770

[B6] Cheng Q. Huang C. Cao H. Lin J. Gong X. Li J. (2019). A novel prognostic signature of transcription factors for the prediction in patients with GBM. *Front. Genet.* 10:906. 10.3389/fgene.2019.00906 31632439 PMC6779830

[B7] Cui D. Huang Z. Liu Y. Ouyang G. (2017). The multifaceted role of periostin in priming the tumor microenvironments for tumor progression. *Cell Mol. Life Sci.* 74 4287–4291. 10.1007/s00018-017-2646-2 28884337 PMC11107730

[B8] Dagogo-Jack I. Shaw A. T. (2018). Tumour heterogeneity and resistance to cancer therapies. *Nat. Rev. Clin. Oncol.* 15 81–94. 10.1038/nrclinonc.2017.166 29115304

[B9] Das M. Zhu C. Kuchroo V. K. (2017). Tim-3 and its role in regulating anti-tumor immunity. *Immunol. Rev.* 276 97–111. 10.1111/imr.12520 28258697 PMC5512889

[B10] Field C. S. Hunn M. K. Ferguson P. M. Ruedl C. Ancelet L. R. Hermans I. F. (2017). Blocking CTLA-4 while priming with a whole cell vaccine reshapes the oligoclonal T cell infiltrate and eradicates tumors in an orthotopic glioma model. *Oncoimmunology* 7:e1376154. 10.1080/2162402X.2017.1376154 29296535 PMC5739554

[B11] Gieryng A. Pszczolkowska D. Walentynowicz K. A. Rajan W. D. Kaminska B. (2017). Immune microenvironment of gliomas. *Lab Invest.* 97 498–518. 10.1038/labinvest.2017.19 28287634

[B12] GTEx Consortium (2015). Human genomics. The genotype-tissue expression (GTEx) pilot analysis: multitissue gene regulation in humans. *Science* 348 648–660. 10.1126/science.1262110 25954001 PMC4547484

[B13] Hambardzumyan D. Gutmann D. H. Kettenmann H. (2016). The role of microglia and macrophages in glioma maintenance and progression. *Nat. Neurosci.* 19 20–27. 10.1038/nn.4185 26713745 PMC4876023

[B14] Hanahan D. Coussens L. M. (2012). Accessories to the crime: functions of cells recruited to the tumor microenvironment. *Cancer Cell* 21 309–322. 10.1016/j.ccr.2012.02.022 22439926

[B15] Hanahan D. Weinberg R. A. (2011). Hallmarks of cancer: the next generation. *Cell* 144 646–674. 10.1016/j.cell.2011.02.013 21376230

[B16] Hirose T. Virnicchi G. Tanigawa A. Naganuma T. Li R. Kimura H. (2014). NEAT1 long noncoding RNA regulates transcription via protein sequestration within subnuclear bodies. *Mol. Biol. Cell* 25 169–183. 10.1091/mbc.E13-09-0558 24173718 PMC3873887

[B17] Hu G. Gong A. Y. Wang Y. Ma S. Chen X. Chen J. (2016). LincRNA-Cox2 promotes late inflammatory gene transcription in macrophages through modulating SWI/SNF-mediated chromatin remodeling. *J. Immunol.* 196 2799–2808. 10.4049/jimmunol.1502146 26880762 PMC4779692

[B18] Hu H. Mu Q. Bao Z. Chen Y. Liu Y. Chen J. (2018). Mutational landscape of secondary glioblastoma guides MET-Targeted trial in brain tumor. *Cell* 175 1665.e18–1678.e18. 10.1016/j.cell.2018.09.038 30343896

[B19] Hu Q. Ye Y. Chan L. C. Li Y. Liang K. Lin A. (2019). Oncogenic lncRNA downregulates cancer cell antigen presentation and intrinsic tumor suppression. *Nat. Immunol.* 20 835–851. 10.1038/s41590-019-0400-7 31160797 PMC6619502

[B20] Huang D. Chen J. Yang L. Ouyang Q. Li J. Lao L. (2018). NKILA lncRNA promotes tumor immune evasion by sensitizing T cells to activation-induced cell death. *Nat. Immunol.* 19 1112–1125. 10.1038/s41590-018-0207-y 30224822

[B21] Jackson C. M. Choi J. Lim M. (2019). Mechanisms of immunotherapy resistance: lessons from glioblastoma. *Nat. Immunol.* 20 1100–1109. 10.1038/s41590-019-0433-y 31358997

[B22] Jia Q. Wu W. Wang Y. Alexander P. B. Sun C. Gong Z. (2018). Local mutational diversity drives intratumoral immune heterogeneity in non-small cell lung cancer. *Nat. Commun.* 9:5361. 10.1038/s41467-018-07767-w 30560866 PMC6299138

[B23] Jiang T. Mao Y. Ma W. Mao Q. You Y. Yang X. (2016). CGCG clinical practice guidelines for the management of adult diffuse gliomas. *Cancer Lett.* 375 263–273. 10.1016/j.canlet.2016.01.024 26966000

[B24] Lapointe S. Perry A. Butowski N. A. (2018). Primary brain tumours in adults. *Lancet* 392 432–446. 10.1016/S0140-6736(18)30990-5 30060998

[B25] Li Y. Jiang T. Zhou W. Li J. Li X. Wang Q. (2020). Pan-cancer characterization of immune-related lncRNAs identifies potential oncogenic biomarkers. *Nat. Commun.* 11:1000. 10.1038/s41467-020-14802-2 32081859 PMC7035327

[B26] Louis D. N. Perry A. Reifenberger G. von Deimling A. Figarella-Branger D. Cavenee W. K. (2016). The 2016 world health organization classification of tumors of the central nervous system: a summary. *Acta Neuropathol.* 131 803–820. 10.1007/s00401-016-1545-1 27157931

[B27] Ma B. Li Y. Ren Y. (2020). Identification of a 6-lncRNA prognostic signature based on microarray re-annotation in gastric cancer. *Cancer Med.* 9 335–349. 10.1002/cam4.2621 31743579 PMC6943089

[B28] Ma S. Dai Y. (2011). Principal component analysis based methods in bioinformatics studies. *Brief. Bioinform.* 12 714–722. 10.1093/bib/bbq090 21242203 PMC3220871

[B29] Machulla H. K. Steinborn F. Schaaf A. Heidecke V. Rainov N. G. (2001). Brain glioma and human leukocyte antigens (HLA)- -is there an association. *J. Neurooncol.* 52 253–261. 10.1023/a:1010612327647 11519856

[B30] Malinchoc M. Kamath P. S. Gordon F. D. Peine C. J. Rank J. ter Borg P. C. (2000). A model to predict poor survival in patients undergoing transjugular intrahepatic portosystemic shunts. *Hepatology* 31 864–871. 10.1053/he.2000.5852 10733541

[B31] Mathy N. W. Chen X. M. (2017). Long non-coding RNAs (lncRNAs) and their transcriptional control of inflammatory responses. *J. Biol. Chem.* 292 12375–12382. 10.1074/jbc.R116.760884 28615453 PMC5535013

[B32] Molinaro A. M. Taylor J. W. Wiencke J. K. Wrensch M. R. (2019). Genetic and molecular epidemiology of adult diffuse glioma. *Nat. Rev. Neurol.* 15 405–417. 10.1038/s41582-019-0220-2 31227792 PMC7286557

[B33] Nduom E. K. Weller M. Heimberger A. B. (2015). Immunosuppressive mechanisms in glioblastoma. *Neuro Oncol.* 17(Suppl. 7), vii9–vii14. 10.1093/neuonc/nov151 26516226 PMC4625890

[B34] Newman A. M. Liu C. L. Green M. R. Gentles A. J. Feng W. Xu Y. (2015). Robust enumeration of cell subsets from tissue expression profiles. *Nat. Methods* 12 453–457. 10.1038/nmeth.3337 25822800 PMC4739640

[B35] Ostrom Q. T. Cioffi G. Gittleman H. Patil N. Waite K. Kruchko C. (2019). CBTRUS statistical report: primary brain and other central nervous system tumors diagnosed in the United States in 2012-2016. *Neuro Oncol.* 21(Suppl. 5), v1–v100. 10.1093/neuonc/noz150 31675094 PMC6823730

[B36] Peng L. Chen Z. Chen Y. Wang X. Tang N. (2019). MIR155HG is a prognostic biomarker and associated with immune infiltration and immune checkpoint molecules expression in multiple cancers. *Cancer Med.* 8 7161–7173. 10.1002/cam4.2583 31568700 PMC6885872

[B37] Peng Z. Liu C. Wu M. (2018). New insights into long noncoding RNAs and their roles in glioma. *Mol Cancer* 17:61. 10.1186/s12943-018-0812-2 29458374 PMC5817731

[B38] Pereira B. I. Devine O. P. Vukmanovic-Stejic M. Chambers E. S. Subramanian P. Patel N. (2019). Senescent cells evade immune clearance via HLA-E-mediated NK and CD8+ T cell inhibition. *Nat. Commun.* 10:2387. 10.1038/s41467-019-10335-5 31160572 PMC6547655

[B39] Pitt J. M. Marabelle A. Eggermont A. Soria J. C. Kroemer G. Zitvogel L. (2016). Targeting the tumor microenvironment: removing obstruction to anticancer immune responses and immunotherapy. *Ann. Oncol.* 27 1482–1492. 10.1093/annonc/mdw168 27069014

[B40] Poon C. C. Sarkar S. Yong V. W. Kelly J. J. P. (2017). Glioblastoma-associated microglia and macrophages: targets for therapies to improve prognosis. *Brain* 140 1548–1560. 10.1093/brain/aww355 28334886

[B41] Qian J. Wang C. Wang B. Yang J. Wang Y. Luo F. (2018). The IFN-γ/PD-L1 axis between T cells and tumor microenvironment: hints for glioma anti-PD-1/PD-L1 therapy. *J. Neuroinflammation* 15:290. 10.1186/s12974-018-1330-2 30333036 PMC6192101

[B42] Quail D. F. Joyce J. A. (2017). The Microenvironmental Landscape of Brain Tumors. *Cancer Cell* 31 326–341. 10.1016/j.ccell.2017.02.009 28292436 PMC5424263

[B43] Serratì S. De Summa S. Pilato B. Petriella D. Lacalamita R. Tommasi S. (2016). Next-generation sequencing: advances and applications in cancer diagnosis. *Onco Targets Ther.* 9 7355–7365. 10.2147/OTT.S99807 27980425 PMC5144906

[B44] Sturm D. Pfister S. M. Jones D. T. W. (2017). Pediatric gliomas: current concepts on diagnosis. biology, and clinical management. *J. Clin. Oncol.* 35 2370–2377. 10.1200/JCO.2017.73.0242 28640698

[B45] Tan A. C. Ashley D. M. López G. Y. Malinzak M. Friedman H. S. Khasraw M. (2020). Management of glioblastoma: state of the art and future directions. *CA Cancer J. Clin.* 70 299–312. 10.3322/caac.21613 32478924

[B46] Topalian S. L. Taube J. M. Anders R. A. Pardoll D. M. (2016). Mechanism-driven biomarkers to guide immune checkpoint blockade in cancer therapy. *Nat. Rev. Cancer* 16 275–287. 10.1038/nrc.2016.36 27079802 PMC5381938

[B47] Wang D. Li J. Cai F. Xu Z. Li L. Zhu H. (2019). Overexpression of MAPT-AS1 is associated with better patient survival in breast cancer. *Biochem. Cell Biol.* 97 158–164. 10.1139/bcb-2018-0039 30074401

[B48] Wang F. Cao X. Yin L. Wang Q. He Z. (2020). Identification of SCARA5 gene as a potential immune-related biomarker for triple-negative breast cancer by integrated analysis. *DNA Cell Biol.* 39 1813–1824. 10.1089/dna.2020.5449 32816580

[B49] Xu M. Xu X. Pan B. Chen X. Lin K. Zeng K. (2019). LncRNA SATB2-AS1 inhibits tumor metastasis and affects the tumor immune cell microenvironment in colorectal cancer by regulating SATB2. *Mol. Cancer* 18:135. 10.1186/s12943-019-1063-6 31492160 PMC6729021

[B50] Yoshihara K. Shahmoradgoli M. Martínez E. Vegesna R. Kim H. Torres-Garcia W. (2013). Inferring tumour purity and stromal and immune cell admixture from expression data. *Nat. Commun.* 4:2612. 10.1038/ncomms3612 24113773 PMC3826632

[B51] Zhang M. Sun L. Ru Y. Zhang S. Miao J. Guo P. (2020a). A risk score system based on DNA methylation levels and a nomogram survival model for lung squamous cell carcinoma. *Int. J. Mol. Med.* 46 252–264. 10.3892/ijmm.2020.4590 32377703 PMC7255475

[B52] Zhang M. Wang X. Chen X. Zhang Q. Hong J. (2020b). Novel immune-related gene signature for risk stratification and prognosis of survival in lower-grade glioma. *Front. Genet.* 11:363. 10.3389/fgene.2020.00363 32351547 PMC7174786

