# Supplementary figures and images for: An Immune Gene-Related Five-lncRNA Signature for to Predict Glioma Prognosis
Source: Front Genet. 2020 Dec 16;11:612037. doi: 10.3389/fgene.2020.612037 (PMC7772413; doi:10.3389/fgene.2020.612037)

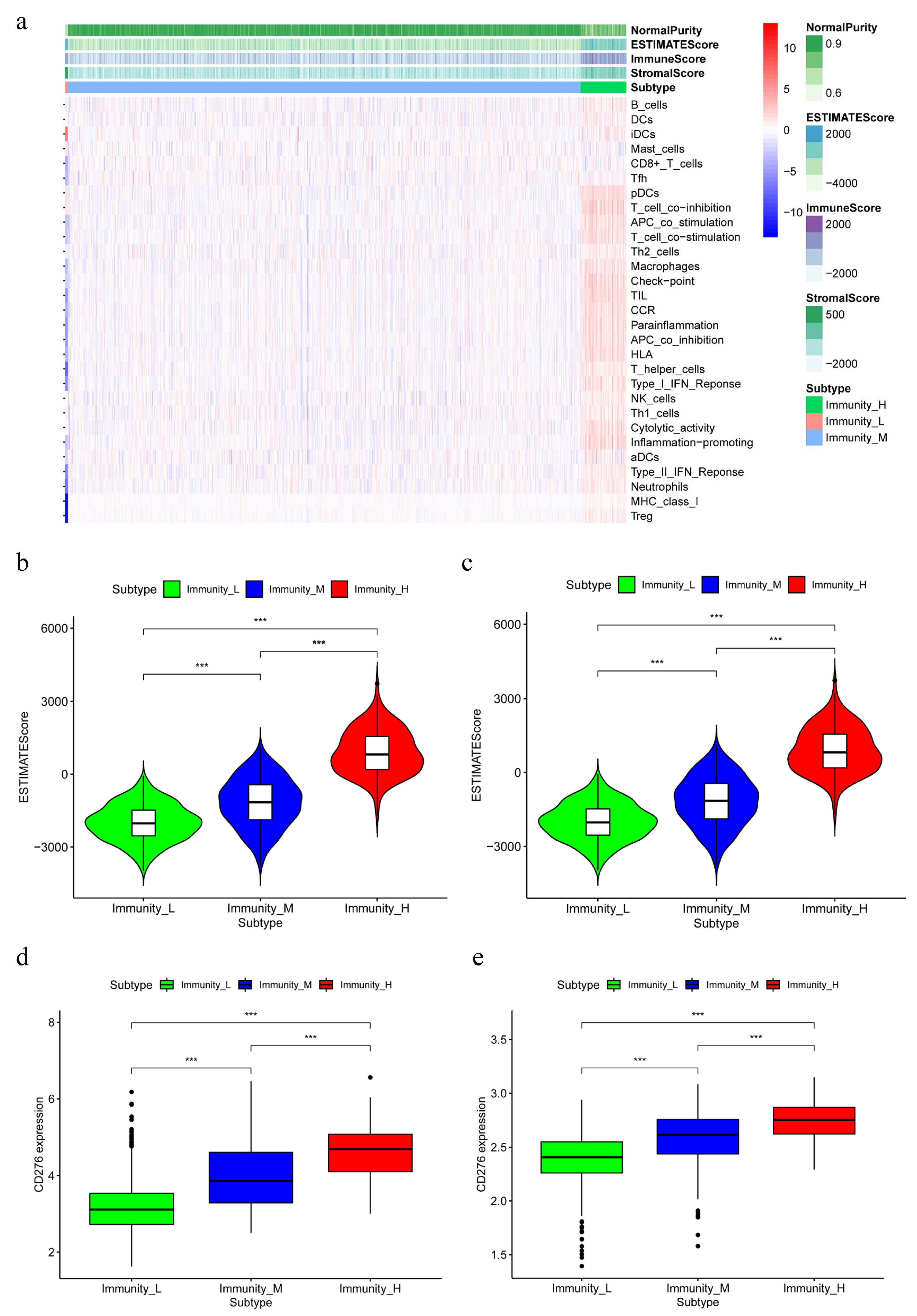

Supplement: Supplementary Figure 1 — (A) The heatmap of the immune microenvironment of normal brain tissue base on GTEX. The relationship between immune grouping and ESTIMATEScore in TCGA (B) and CGGA (C). The correlation between CD276 and immune grouping in TCGA (D) and CGGA (E). [file Figure_1.TIF]

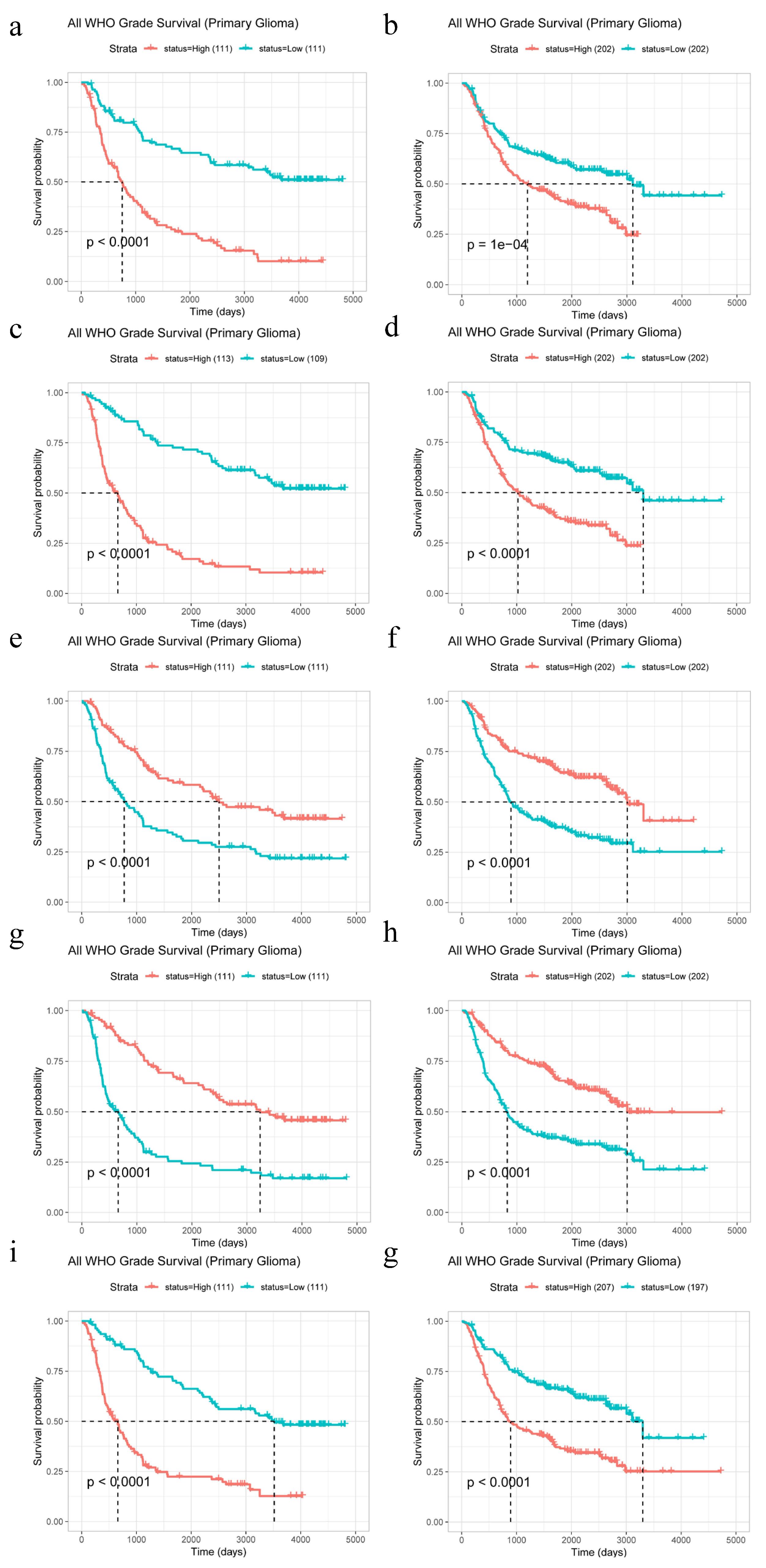

Supplement: Supplementary Figure 2 — Survival curve of glioma patients in CGGA mRNAseq_693 and CGGA mRNAseq_325. (A) ap0001007.1, (C) LBX2-AS1, (E) LINC00515, (G) MAPT-AS1, (I) MIR155HG in CGGA mRNAseq_325. (B) ap0001007.1, (D) LBX2-AS1, (F) LINC00515, (H) MAPT-AS1, (G) MIR155HG in CGGA mRNAseq_325 database. [file Figure_2.TIF]

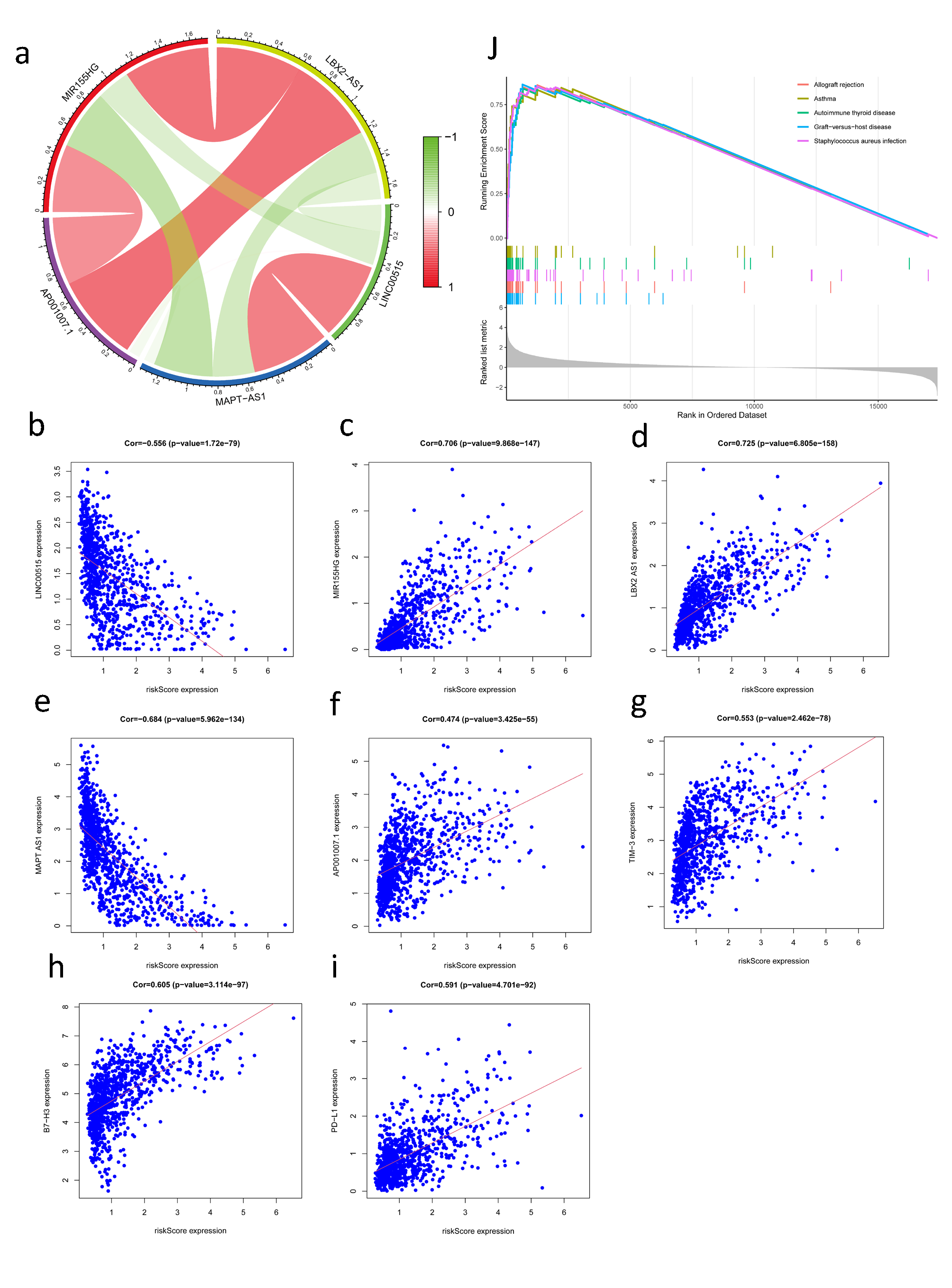

Supplement: Supplementary Figure 3 — Correlation analysis of risk score, lncRNA, and immune checkpoint. (A) Correlation analysis of lncRNAs related to immune gene set. (B) KEGG in CGGA. (C) Analysis of the correlation between risk scores and lncRNAs or immune checkpoints in (B) LINC00515, (C) MIR155HG, (D) LBX2-AS1, (E) MAPT-AS1, (F) ap0001007.1 or (G) TIM-3, (H) B7-H3, (I) PDL1, (J) KEGG in CGGA. [file Figure_3.tif]
