# Supplementary material for: An Immune Gene-Related Five-lncRNA Signature for to Predict Glioma Prognosis
Source: Front Genet. 2020 Dec 16;11:612037. doi: 10.3389/fgene.2020.612037 (PMC7772413; doi:10.3389/fgene.2020.612037)
Supplement: Supplementary file 4 [file Table_1.DOCX]

| Supplementary Table 1：Primer sequence used in this study | | |
| --- | --- | --- |
| LINLBX2-AS1 | Forward primer | GGCATGGCATACAGACAAGG |
|  | reverse primer | GCAAGGGCAACTTCAAGGAA |
| MIR155HG | Forward primer | AGATGGCTCTAATGGTGGCA |
|  | reverse primer | ACAGCATACAGCCTACAGCA |
| MAPT-AS1 | Forward primer | ACCAGAAGGGAGGGATTTGG |
|  | reverse primer | TGCAGAGACTGGAGTGACAG |
| LINC00515 | Forward primer | TGTTGAACGGAGCAGTGATG |
|  | reverse primer | CTAAGCAAAGCCTCCACACC |
| AP001007.1 | Forward primer | TGCTGTAAGGAAAGGGCTCA |
|  | reverse primer | CAGCCACCACATCCAGAGTA |
| PD-L1 | Forward primer | GCTGAATTGGTCATCCCAGAA |
|  | reverse primer | CAGTGCTACACCAAGGCATAA |
| CTLA4 | Forward primer | GTGAACCTCACTATCCAAGGAC |
|  | reverse primer | TGCCTATGCCCAGGTAGTA |
| CD3 | Forward primer | CAGAATTGGAGCAAAGTGGTTATT |
|  | reverse primer | CTCTTGCCCTCAGGTAGAGATA |
| CD8 | Forward primer | CCTTCTCCTGTCACTGGTTATC |
|  | reverse primer | GGCTTGTCTCCCGATTTGA |
| INOS | Forward primer | GTCAGAGTCACCATCCTCTTTG |
|  | reverse primer | GCAGCTCAGCCTGTACTTATC |
| GAPDH | Forward primer | ACCACAGTCCATGCCATCAC |
|  | reverse primer | TCCACCACCCTGTTGCTGTA |
